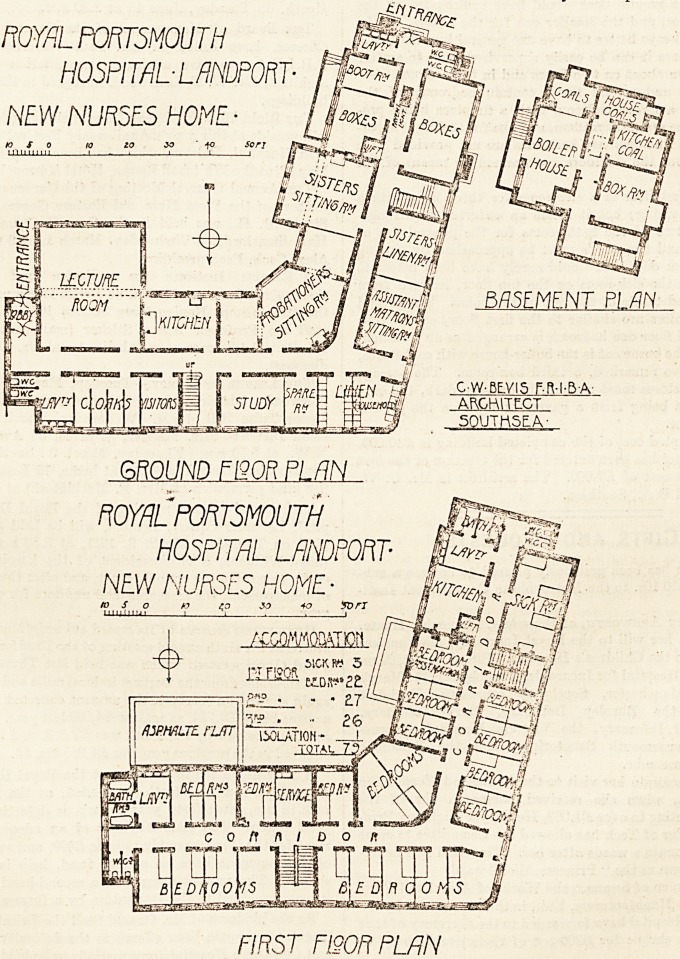# Royal Portsmouth Hospital

**Published:** 1911-03-04

**Authors:** 


### ROYAL PORTSMOUTH HOSPITAL.

### NEW NURSES' HOME.

THIS building, part only of which is in course of erection, is placed in the north-west corner of the site, and is planned in the form of an L with a rather obtuse angle. Accommodation is provided for seventy-five nurses, and in addition there is a sick room for three beds, and an isolation room for one bed, making a total of seventy-nine beds.

On the ground floor there are two entrances, one at either end, and two staircases, not at the extreme ends, but well separated from each other. A common sitting-room is provided for sisters, and another for probationers. The latter is a large room and only one fireplace is shown, but we presume the additional heating power is provided in the shape of radiators, as it would be wholly impossible to warm so large a room with only one fireplace. Apparently the sisters' room can be thrown *en suite* with the probationers' room if necessary. There is a large lecture-room with kitchen adjoining. The kitchen, we imagine, is intended as a practice kitchen for the nurses. A small study is also provided and a room for visitors; next to the study is a spare room, the use of which is not apparent. Neither of these three rooms have fireplaces. There is a sitting-room for the assistant matron, and a sisters' linen-room adjoining. The latter is a provision we have never seen before and do not understand the meaning of; in addition there are two other linen-rooms. Two box-rooms are provided and a boot-room. There is also a third box-room in the basement. We should have thought that the large box-room on the ground floor would have sufficed for all the nurses' boxes, and the smaller one for the sisters. It is, of course, always better to have one general box-room in a position where it can be easily supervised than to scatter the provision about on two floors and in three rooms.

The first and second floors contain bedrooms of the usual type, a larger bedroom with a fireplace being provided for the assistant matron, and one for the night superintendent. The ordinary bedrooms are not provided with fireplaces, but the corridors are warmed by means of hot water.

On the first floor is a sick-room for three beds with a kitchen adjoining; but it seems an unfortunate arrangement that two of the bath-rooms for the general use of the nurses and two w.c.'s must be approached by passing the sick-room door. It would surely have been better to have placed the sick-room on the top floor, isolated from the noise and traffic of the rest of the home. The second and third floors are similar to the first floor, except that on the third floor one bedroom is arranged as an isolation-room. In the basement is the boiler-house with coal stores, and, as above remarked, a third box-room. The passage to the coal stores must necessarily be very dark, the only light it gets being from a pavement light on the ground floor corridor.

The estimated cost of the completed building is £10,000, and a contract has been entered for the erection of the first portion at a cost of £7,000. The architect is Mr. C. W. Bevis, F.R.I.B.A., Southsea.

## Figures and Tables

**Figure f1:**